# A study on differences about the influencing factors of depressive symptoms between medical staff and residents during 2022 city-wide temporary static management period to fighting against COVID-19 pandemic in Shanghai

**DOI:** 10.3389/fpubh.2022.1083144

**Published:** 2023-01-09

**Authors:** Ying Zhao, Yiran Tao, Xiwen Bao, Qiang Ding, Changyan Han, Tingkun Luo, Weijia Zhang, Jinhua Sun, Jiali Shi

**Affiliations:** ^1^Department of Psychological Medicine, Children's Hospital of Fudan University, Shanghai, China; ^2^Department of General Medicine, Zhoupu Health Service Center, Pudong New Area, Shanghai, China; ^3^Shanghai University of Medicine and Health Science Affiliated Zhoupu Hospital, Shanghai, China; ^4^Department of Psychiatry, Tongji University Affiliated Shanghai Pudong New Area Mental Health Center, Shanghai, China

**Keywords:** COVID-19, depressive symptoms, latent class analysis, medical staff, residents

## Abstract

**Objectives:**

Our study aimed to identify the latent class of depressive symptoms in the Shanghai population during the city-wide temporary static management period and compare differences in the factors influencing depressive symptoms between medical staff and residents.

**Methods:**

An online cross-sectional survey was conducted with 840 participants using questionnaires, including Patient Health Questionnaire-9 (PHQ-9), Generalized Anxiety Disorder-7 (GAD-7), Pittsburgh Sleep Quality Index (PSQI), and self-compiled questionnaire (demographic characteristics and internet usage time). Latent class analysis (LCA) was performed based on participants' depressive symptoms. The latent class subgroups were compared using the chi-square test and *t-*test. Logistic regression was used in our study to analyze the factors influencing depressive symptoms within the medical staff group and residents group and then compare their differences.

**Results:**

Two distinct subgroups were identified based on the LCA: the group with low-depressive symptoms and the group with high-depressive symptoms. There were significant differences between the two groups (*P* < 0.05) on age, education level, marital status, internet usage time, identity characteristics (medical staff or residents), family income level, living style, overall quality of sleep, and anxiety levels. Furthermore, logistic regression analysis results showed that compared with the residents group, the participants in the group of medical staff with “increasing internet usage time” and the “daytime dysfunction” would have nearly two times the possibility of getting serious depressive symptoms.

**Conclusions:**

There are differences in the factors influencing depression symptoms between medical staff and residents during the 2022 city-wide temporary static management period to fighting against the COVID-19 pandemic in Shanghai. We should pay special attention to those with increasing internet usage time and daytime dysfunction in medical staff working in a special environment such as the COVID-19 pandemic.

## Introduction

The World Health Organization (WHO) declared a global pandemic of the Coronavirus Disease 2019 (COVID-19) on March 11, 2020 (1). A lockdown policy is one of the most important non-pharmaceutical interventions (NPI) measures to control the spread of COVID-19 (2). As one of the biggest cities in China, Shanghai announced a city-wide temporary static management (all citizens must be stationary in their location, limitation of movement) in March 2022. Temporary static management for more than 2 months reduced unnecessary face-to-face social contact, finally becoming the most powerful intervention to control the pandemic by limiting the spread of infectious sources. However, a sudden cessation of interpersonal communication has changed common daily lifestyles, and people under static management had to adapt to sudden social isolation. Previous reports showed that changes in daily lifestyle brought about by COVID-19 led to a higher tendency of severe emotional distress and depression. These psychological consequences not only exist for a short time during the pandemic period but may also extend for several months after the infection.

There are many scales and questionnaires for assessing the psychological state including the assessment of the anxiety and depression level of the affected people suffered these disastrous events. The Patient Health Questionnaire-9 (PHQ-9) is a widely used screening tool for depression episodes. The cut-off point for PHQ-9 is 10 points which could identify high-risk individuals for depression episodes (3). Yet trajectories of depressive episodes are complicated and might originate from the interaction of internal and external factors, including genetic, psychological, and environmental risk factors (4). Early screening of depressive symptoms by cut-off value may decrease the sensitivity of the screening test. Consequently, a subgroup of individuals with suspected depressive symptoms (below the cut-off value) would have been ignored, especially under city-wide static management. Latent class analysis (LCA) is a robust probabilistic approach which bases on the characteristics of the data. LCA can provide a more sensitive and effective classification to identify the subpopulation of individuals with a potential for depression episodes whose PHQ-9 scores are under 10 points cut-off (5).

Under the circumstance of this sudden static management, many complicated risk factors affected the occurrence and development of depression. A depression and anxiety online survey in America during the COVID-19 pandemic showed that males were 1.42 times more likely to suffer from depression than females. In addition, Hispanics (2.52 times higher), medical staff (2.40 times higher), those surrounded with their children at home (1.42 times higher), and those with income <US $60,000 (1.43 times higher) had higher odds of depression (6). Furthermore, an extended number of days in quarantine and lack of physical exercise were associated with increased depression (7). Recent studies found that loneliness strongly predicted depressive symptoms during COVID-19-related static management (8). However, the background of these previous studies was not based on thorough city-wide static management.

Noteworthily, unlike residents stuck at home to fight against COVID-19 pandemic, frontline medical staff had to isolate themselves from their families, experience physical exhaustion, loneliness, and panic of uncertainty for nearly 2 months. They have high workloads and long working hours, high risk of infection (9), social stigmatization, concern about spreading the virus to their families (10, 11) and lack of more social contact (12). The COVID-19 pandemic has had a significant psychological impact on health professionals (13, 14), so the factors influencing depressive symptoms in medical staff may differ from those for residents. Our study was designed to identify the latent class of depression symptoms in Shanghai population during the city-wide temporary static management, and investigate and compare the difference in the factors influencing depressive symptoms between front-line medical staff and the residents.

## Materials and methods

### Participants

This was a web-based survey study and implemented across Shanghai from April 4 to June 3, 2022.

This study used a snowball non-probability/convenience sampling method. Although random sampling was used in the initial selection of survey subjects, the final samples were all non-probability samples, so non-probability sampling was adopted. The questionnaires were sent to the participants via a survey APP (a questionnaire application called “wenjuanxing”). The response of the participants in questionnaires was automatically saved in APP. This study was approved by the ethics committee of the Children's Hospital of Fudan University, Shanghai and was complied with the Declaration of Helsinki.

In 2 months, a total of 840 responses were received. Participation in the survey was voluntary, and confidentiality of the responses was ensured.

### Measures

The survey consists of four parts: depressive symptoms, anxiety, sleep quality, and a self-compiled questionnaire (demographic characteristics and whether participants have noticed an increase in internet usage time). We assessed anxiety, sleep quality, and depressive symptoms for all participants using the Chinese versions of PHQ-9, Generalized Anxiety Disorder-7 (GAD-7), and Pittsburgh Sleep Quality Index (PSQI), which have good validity and reliability as stated below.

PHQ-9 is widely used to screen for depressive symptoms and assess the severity of depressive symptoms in the population. It contains nine items, each based on the duration of depressive symptoms over a 2-week period. Each item is scored as follows: 0 = not at all; 1 = several days; 2 = more than half of all the days; 3 = nearly every day; The scores of the nine items are added, and higher total scores indicate more severe depression symptoms. The Cronbach's alpha for this questionnaire was 0.80 (3).

GAD-7 mainly assesses the severity of anxiety symptoms in the past 2 weeks, including seven items covering the main feelings and physical discomforts of anxiety symptoms. According to the duration of anxiety symptoms, GAD-7 is divided into 0–3 grades: 0, Not at all; 1, Some days; 2, More than half the days; and 3, Nearly every day for scoring. The total score is the sum of seven items. The higher the total score, the more severe the anxiety symptoms (15). The Cronbach's alpha for this questionnaire was 0.92 (16).

PSQI has been widely used in the survey of sleep quality in various populations. PSQI contains 19 self-reported items that constitute seven aspects of sleep problems, including sleep latency, sleep duration, habitual sleep efficiency, sleep disturbance, subjective sleep quality, hypnotic drugs, and daytime function, and each dimension is scored 0–3 points. The sum of the seven component scores is the global score of PSQI (0–21 points). Higher total PSQI scores indicate worse sleep quality. The Cronbach's alpha for this questionnaire was 0.75 (17).

### Statistical analysis

Data analysis was performed with R version 4.1.3. The method of multiple imputations was employed on variables with no more than 20% missing value, and the predictive mean matching (PMM) method was adopted using the “mice” package (18).

The normality of the data was analyzed using the Kolmogorov–Smirnov test and Q-Q plots. Normally distributed variables were expressed as the mean (standard deviation [SD]), while non-normally distributed variables were expressed as the median (interquartile range [IQR]). Categorical variables were expressed as frequencies (n) and percentages (%).

The “poLCA” package (19) in R was used for latent class analysis. The PHQ9 items were recoded into binary variables for the LCA. Items with a score of 1 were denoted as 0, and a score of 2 or more as 1. An exploratory approach was adopted starting from a two-class model, and the analysis was performed by increasing the number of classes. Model fit indices (the Akaike information criterion (AIC), Bayesian information criterion (BIC), and Maximum Log-likelihood) were used to evaluate the best model. A low value for AIC and BIC or a high value for Maximum Log-likelihood indicated a better model (20). In addition, the entropy (21), which indicates the degree of accuracy of the model that defines the classes, was employed to select the most optimal model. In this study, the posterior probability was used as the index of certainty classification. The posterior probability represents the probability that a person will be assigned to the high or low depressive symptom group based on the severity of their depressive symptoms. The value of the best-fit class is close to 1, meanwhile the value of the other classes is close to 0, indicating a higher certainty of classification. When the posterior probability of the model is <90%, we ceased adding a class to fit the model (22).

To describe the characteristics of different latent classes, the *t-*test was used for normally distributed continuous variables, the Wilcoxon test was used for non-normally distributed continuous variables, and the chi-square test was used for categorical variables. We used boxplots to show differences in total PSQI score and total GAD-7 score among latent classes.

With the latent category groupings as the dependent variable, and multiple factors influencing depressive symptoms as independent variables, univariate logistic regression analyses were conducted separately in the medical staff group and residents group to examine variables associated with depressive symptoms.

## Results

### Characteristics of the participants

A sum of 840 participants were investigated, including 120 medical staff and 720 residents. Table 1 presents the baseline characteristics of the whole participants and participants with different identities, including medical staff or residents. The median age of all the participants was 40.0 years (SD = 12.14), and 302 (36.0%) were males.

**Table 1:** Characteristics of participants.

**Variables**	**All participants** ** (*N =* 840)**	**Medical staff** ** (*N =* 120)**	**Residents ** **(*N =* 720)**	***P*-value**
**Age**	40.00 ± 12.14	32.77 ± 7.76	41.14 ± 12.32	< 0.001^**^
**Gender**				0.076
Male	302 (36.0)	34 (28.3)	268 (37.2)	
Female	538 (64.0)	86 (71.7)	452 (62.8)	
**Education (with bachelor's degree or higher)**				< 0.001^**^
Yes	578 (68.8)	118 (98.3)	460 (63.9)	
No	262 (31.2)	2 (0.7)	260 (36.1)	
**Married**				0.001^*^
Yes	591 (70.4)	68 (56.7)	523 (72.6)	
No	249 (29.6)	52 (43.3)	197 (27.4)	
**Increasing internet usage time**				0.458
Yes	266 (31.7)	42 (35.0)	224 (31.1)	
No	574 (68.7)	78 (65.0)	496 (68.9)	
**Monthly income**				0.069
Low-income (< 10,000 RMB)	575 (68.5)	76 (63.3)	499 (69.3)	
Middle-income (10,000–30,000 RMB)	231 (27.5)	42 (35.0)	189 (26.2)	
High-income (>30,000 RMB)	34 (4.0)	2 (1.7)	32 (4.4)	
**Living Style**				< 0.001^**^
Alone	131 (15.6)	34 (28.3)	97 (13.5)	
With family	634 (75.5)	57 (47.5)	577 (80.1)	
In the company	75 (8.9)	29 (24.2)	46 (6.4)	
**Total score of GAD-7**	3.00 [0.00, 6.00]	4.00 [0.75, 6.00]	2.00 [0.00, 6.00]	0.033^*^
**Total score of PSQI**	5.00 [3.00, 8.00]	6.00 [3.75, 8.00]	5.00 [3.00, 8.00]	0.223
**Total score of PHQ-9**	4.50 [1.00, 8.00]	6.00 [2.00, 9.00]	4.00 [1.00, 8.00]	0.005^*^

* p<0.05;

** p<0.001.

GAD-7, Generalized Anxiety Disorder-7; PSQI, Pittsburgh Sleep Quality Index; PHQ-9, The Patient Health Questionnaire-9; RMB, Chinese Yuan.

A total of 578 participants (68.8%) had a bachelor's degree or higher, 591 (70.4%) were currently married, 575 (68.5%) had lower-middle monthly incomes (<10,000 RMB, Chinese Yuan), 634 (75.5%) were living with family, and 266 (31.7%) reported increasing internet usage time. The median total PSQI and PHQ-9 scores were 5 and 4.5, respectively. There were differences in age, education level, marital status, living style, total GAD-7 score, and total PHQ-9 score between medical staff group and residents group (*P* < 0.05).

### Model fit indices of LCA

Model fit indices for models with different latent classes are listed in Table 2. LCA with 1–8 classes was applied. The results indicated that the AIC and BIC decreased as the classification number increased, the Maximum Log-likelihood increased with an increasing classification number, and the two-class model had the highest entropy value (0.88). Additionally, the posterior probability of each class was >90% only in the two-class model. Other models had posterior probabilities <90%. Therefore, we selected the two-class model to maximize the accuracy of the latent class.

**Table 2:** Fit statistics for latent class models from two to eight classes.

**Model**	**Maximum Log-likelihood**	**AIC**	**BIC**	**Entropy**
1-class	−4,742	9,502	9,545	NA
2-class	−3,643	7,325	7,415	0.88
3-class	−3,472	7,003	7,140	0.81
4-class	−3,439	6,956	7,141	0.76
5-class	−3,431	6,960	7,192	NA
6-class	−3,421	6,960	7,239	NA
7-class	−3,414	6,966	7,293	NA
8-class	−3,402	6,962	7,336	NA

AIC, Akaike information criterion; BIC, Bayesian information criterion.

### Definition of LCA

**Figure 1:**
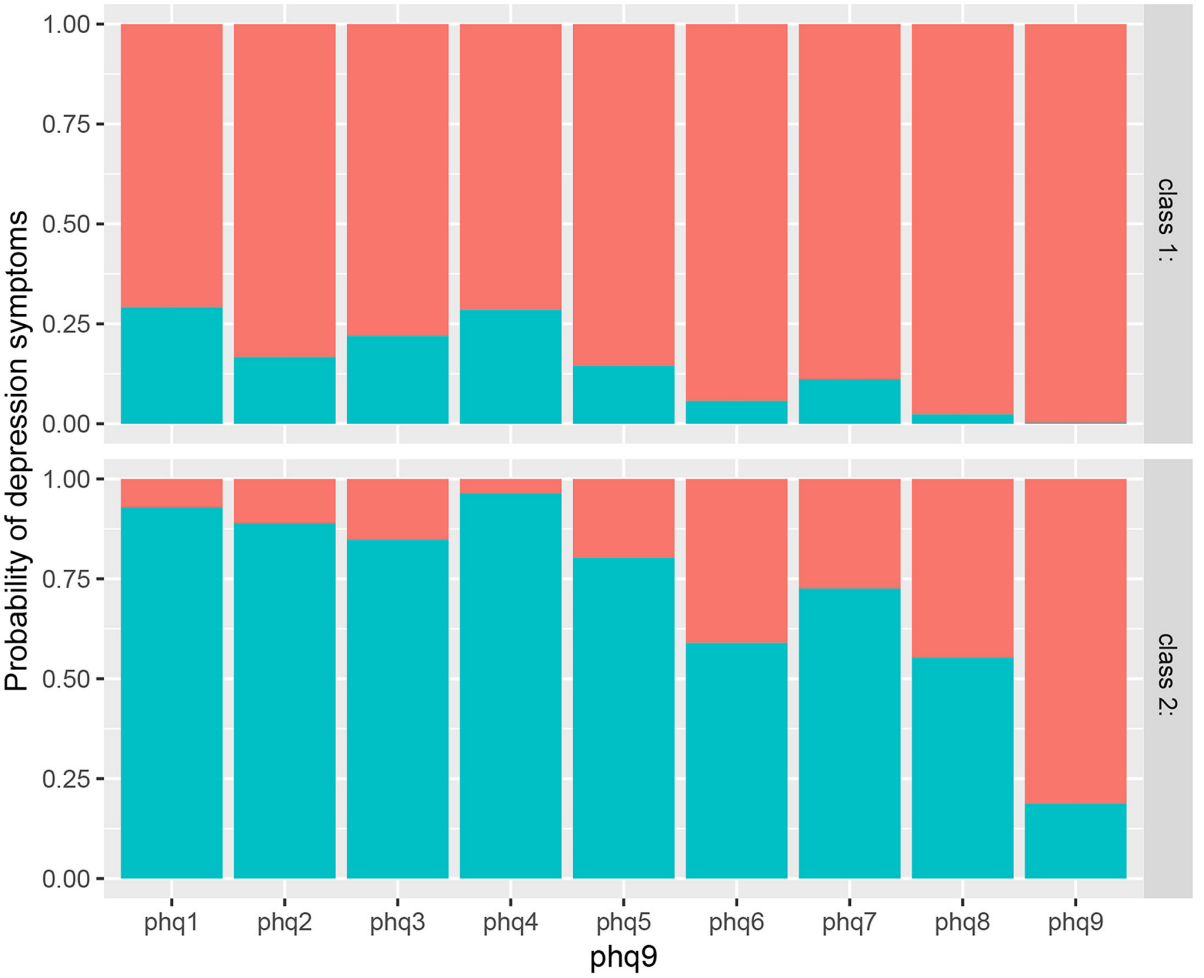
Response probability values of latent classes of depressive symptoms in participants.

As shown in Figure 1, the response probabilities of participants in class 1 were low, indicating that they had better mental health status in the static management and had a potential to effectively regulate their inner mental health, so they were labeled as the low depressive symptoms group.

In contrast, the response probabilities of those in class 2 were high, indicating that these participants had poorer mental health status during the static management and could not effectively regulate and control emotions, so they were labeled as the high depressive symptoms group.

### Comparison of characteristics of the participants between high and low depressive symptoms groups

**Figure 2:**
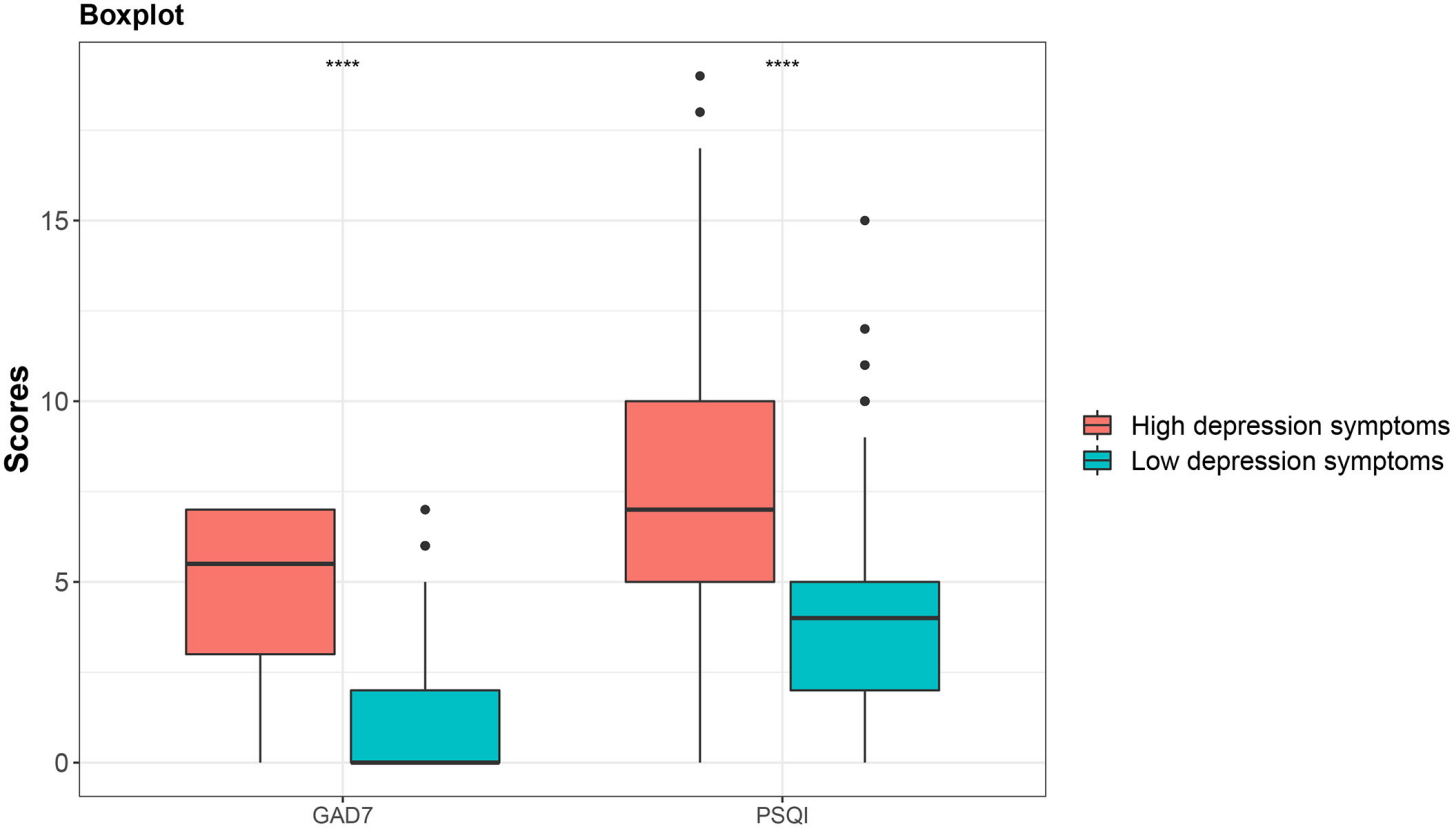
Comparison of total score of GAD7 and total score of PSQI between the low depressive symptoms group and high depressive symptoms group.

As shown in Table 3, age, education level, marital status, internet usage time (whether increased), identity characteristics (medical staff or residents), family income level, and living style were significantly different in the low depressive symptoms group and high depressive symptoms group (*P* < 0.05). In addition, the total GAD-7 and PSQI scores were significantly different between the two groups (Figure 2). Furthermore, the score of each subscale of PSQI was also significantly different between the two groups (Table 4).

**Table 3:** Comparison of demographic characteristics between two latent groups.

**Variables**	**Low depressive symptoms group** ** (*N =* 377)**	**High depressive symptoms group** ** (*N =* 463)**	***P*-value**
Age	43.47 ± 13.11	37.08 ± 10.45	< 0.001^**^
Gender (male/female)	137/240	165/298	0.890
Education (with bachelor's degree or higher) (yes/no)	224/153	354/109	< 0.001^**^
Married (yes/no)	280/97	311/152	0.030^*^
Increasing internet usage time (yes/no)	86/291	180/283	< 0.001^**^
**Identity characteristics**			0.014^*^
Medical staff	41 (10.9)	79(17.1)	
Residents	336 (89.1)	384(82.9)	
**Monthly income**			0.081
Low-income (< 10,000 RMB)	273 (72.4)	302 (65.2)	
Middle-income (10,000–30,000 RMB)	90 (23.9)	141 (30.5)	
High-income (>30,000 RMB)	14 (3.7)	20 (4.3)	
**Living style**			0.002^*^
Alone	52 (13.8)	79 (17.1)	
With family	304 (80.6)	330 (71.3)	
In the company	21 (5.6)	54 (11.7)	

* p<0.05;

** p<0.001.

RMB, Chinese Yuan.

**Table 4:** Comparison of each PSQI subscale among two latent groups.

**Variables**	**Low depressive symptoms group (*N =* 377)**	**high depressive symptoms group (*N =* 463)**	***P*-value**
**Subjective sleep quality**			< 0.001^**^
0	121 (32.1)	28 (6.0)	
1	222 (58.9)	193 (41.7)	
2	34 (9.0)	187 (40.4)	
3	0 (0.0)	55 (11.9)	
**Sleep latency**			< 0.001^**^
0	188 (49.9)	129 (27.9)	
1	141 (37.4)	178 (38.4)	
2	42 (11.1)	95 (20.5)	
3	6 (1.6)	61 (13.2)	
**Sleep duration**			< 0.001^**^
0	251 (66.6)	278 (60.0)	
1	74 (19.6)	62 (13.4)	
2	41 (10.9)	76 (16.4)	
3	11 (2.9)	47 (10.2)	
**Habitual sleep efficiency**			< 0.001^**^
0	253 (67.1)	269 (58.1)	
1	78 (20.7)	117 (25.3)	
**2**	24 (6.4)	30 (6.5)	
**3**	22 (5.8)	47 (10.2)	
**Sleep disturbance**			< 0.001^**^
0	106 (28.1)	13 (2.8)	
1	255 (67.6)	300 (64.8)	
2	16 (4.2)	136 (29.4)	
3	0 (0.0)	14 (3.0)	
**Sleeping medication**			< 0.001^**^
0	370 (98.1)	418 (90.3)	
1	3 (0.8)	18 (3.9)	
2	2 (0.5)	13 (2.8)	
3	2 (0.5)	14 (3.0)	
**Daytime dysfunction**			< 0.001^**^
0	185 (49.1)	26 (5.6)	
1	126 (33.4)	152 (32.8)	
2	58 (15.4)	168 (36.3)	
3	8 (2.1)	117 (25.3)	

** p<0.001.

PSQI, Pittsburgh Sleep Quality Index.

### Logistic regression analysis of latent classes of depressive symptoms in the medical staff group and residents group

Table 5 shows the results of the logistic regression analyses used to calculate odds ratios for variables associated with depressive symptoms in medical staff group and residents group. The category classification was adopted as the dependent variable, and the low depressive symptoms group was used as the reference group.

**Table 5:** Association between variables and depressive symptoms in medical staff group and residents group.

**Variables**	**Medical staff**		**Residents**	
	**OR (95%CI)**	***P*-value**	**OR (95CI)**	***P*-value**
Age	0.99 (0.95–0.05)	0.831	0.95 (0.94–0.97)	< 0.001^**^
Gender (male/female)	1.12 (0.49–2.67)	0.792	0.98 (0.72–0.32)	0.890
Education (with bachelor's degree or higher) (yes/no)	1.95 (0.08-50.18)	0.640	2.11 (1.55–2.88)	< 0.001^**^
Married (yes/no)	1.04 (0.48–2.22)	0.930	0.69 (0.50–0.96)	0.031^*^
Increasing internet usage time (yes/no)	3.86 (1.60–10.45)	0.004^*^	1.97 (1.43–2.74)	< 0.001^*^
**Monthly income**				
Low-income (< 10,000 RMB)	Reference	Reference	Reference	Reference
Middle-income (10,000–30,000 RMB)	0.75 (0.34–0.66)	0.475	1.55 (1.10–2.18)	0.012^*^
High-income (>30,000 RMB)	0.46 (0.02–12.00)	0.590	1.46 (0.71–3.08)	0.311
**Living style**				
Alone	Reference	Reference	Reference	Reference
With family	0.82 (0.33–0.99)	0.665	0.76 (0.49–0.17)	0.217
In the company	1.06 (0.37–3.14)	0.911	2.07 (0.98–4.62)	0.064
**PSQI**				
Subjective sleep quality (A)	5.57 (2.66–11.66)	< 0.001^*^	5.47 (4.11–7.27)	< 0.001^**^
Sleep latency (B)	2.04 (1.25–3.32)	0.004^*^	2.03 (1.69–2.44)	< 0.001^**^
Sleep duration (C)	1.97 (1.18–3.30)	0.010^*^	1.30 (1.11–0.52)	0.001^*^
Habitual sleep efficiency (D)	1.85 (1.05–3.24)	0.032^*^	1.20 (1.02–0.40)	0.028^*^
Sleep disturbance (E)	8.88 (3.17–24.86)	< 0.001^*^	8.17 (5.41–12.34)	< 0.001^**^
Sleeping medication (F)	2.55 (0.85–7.59)	0.093	2.22 (1.37–3.62)	0.001^*^
Daytime dysfunction (G)	6.22 (3.20–12.09)	< 0.001^*^	3.95 (3.19–4.89)	< 0.001^**^
**Total score of GAD-7**	2.41 (1.80–3.24)	< 0.001^*^	2.03 (1.84–2.23)	< 0.001^**^

* p<0.05;

** p<0.001.

GAD-7, Generalized Anxiety Disorder-7; PSQI, Pittsburgh Sleep Quality Index; RMB, Chinese Yuan.

The results showed that compared with the low symptoms group, the medical staff in the high depressive symptoms group were more likely to have increasing internet usage time (OR = 3.86, 95%CI: 1.60-10.45, *P*=0.004), worse subjective sleep quality (OR = 5.57, 95%CI: 2.66–11.66, *P* < 0.001); longer sleep latency (OR = 2.04, 95%CI: 1.25–3.32, *P* < 0.001), shorter sleep duration (OR = 1.97, 95%CI: 1.18–3.30, *P* = 0.010), less habitual sleep efficiency (OR = 2.54, 95%CI: 1.53–4.20, *P* = 0.032), more serious sleep disturbance (OR = 8.88, 95%CI: 3.17–24.86, *P* < 0.001), more severe daytime dysfunction (OR = 6.22, 95%CI: 3.20–12.09, *P* < 0.001), and higher GAD7 score (OR = 2.41, 95%CI: 1.80–3.24, *P* < 0.001).

Residents in high depressive symptoms group were more likely to have good education level (OR = 2.11, 95%CI: 1.55–2.88, *P* < 0.001), increasing internet usage time (OR = 1.97, 95%CI: 1.43–2.74, *P* < 0.001), middle income level (OR = 1.55, 95%CI: 1.10–2.18, *P* = 0.012), worse subjective sleep quality (OR = 5.47, 95%CI: 4.11–7.27, *P* < 0.001), longer sleep latency (OR = 2.03, 95%CI: 1.69–2.44, *P* < 0.001), shorter sleep duration (OR = 1.30, 95%CI: 1.11–1.52, *P* = 0.001), less habitual sleep efficiency (OR = 1.20, 95%CI: 1.02–1.40, *P* = 0.028), more serious sleep disturbance (OR = 8.17, 95%CI: 5.41–12.34, *P* < 0.001), more use of sleeping medication (OR = 2.22, 95%CI: 1.37–3.62, *P* < 0.001), more severe daytime dysfunction (OR = 3.95, 95%CI: 3.19–4.89, *P* < 0.001), and higher GAD7 score (OR = 2.03, 95%CI: 1.84–2.23, *P* < 0.001). In contrast, age (OR = 0.95, 95%CI: 0.94–0.97, *P* < 0.001) and marital status (OR = 0.69, 95%CI: 0.50–0.96, *P* = 0.031) were protective factors for depressive symptoms.

We noticed that, compared with the residents group, the participants in the group of medical staff with “increasing internet usage time” and the “daytime dysfunction” would have nearly two times the possibility of getting serious depressive symptoms. After adjusting the age, gender, education, marriage, monthly income, lifestyle and total score of GAD-7, the participants in the group of medical staff with “increasing internet usage time” and the “daytime dysfunction” would also have nearly two times the possibility of getting serious depressive symptoms (increasing internet usage time: OR = 3.84, 95%CI: 1.58–10.42, *P* < 0.001 VS OR = 1.72, 95%CI: 1.23–2.42, *P* < 0.001; daytime dysfunction: OR = 7.09, 95%CI: 3.72-15.91, *P* < 0.001 VS OR = 3.91, 95%CI: 3.16-4.91, *P* < 0.001).

## Discussion

Understanding the factors influencing depressive symptoms during the city-wide static management period to fight against the COVID-19 pandemic is important for early identification and intervention. As far as we know, this study is the first attempt to investigate the difference in influencing factors of depressive symptoms between medical staff and residents during the 2022 COVID-19 pandemic in Shanghai. A number of studies have shown that COVID-19 pandemic was likely to trigger and aggravate mental health problems, including depressive symptoms (7, 23, 24). We found that during the city-wide static management period for COVID-19, medical staff had higher depressive symptoms than residents.

We used LCA to identify two meaningful classes: the high depression symptoms group (463/840, 55.1%) and low depression symptoms group (377/840, 44.9%). The number of people in the two groups was roughly the same, indicating great differences in the psychological adjustment and adaptability of participants during the pandemic. LCA is a flexible statistical method that aims to detect heterogeneity by analyzing individual behavior patterns and finding common types within the population (25). AIC BIC, Maximum Log-likelihood, entropy, and posterior probability are the most commonly used evaluation indexes in selecting the optimal latent class model. Most previous studies applied no more than three of the five common evaluation indexes (26–28). We selected the optimal model using all five evaluation indexes for a more reliable and comprehensive evaluation.

The analysis of characteristics of low and high depressive symptom groups showed that younger adults, people with higher education levels, married participants, increasing internet usage time, medical staff, and those living away from family were prone to experience more severe depressive symptoms. Previous evidence showed similar results that younger people, women, individuals with lower educational and socioeconomic backgrounds, and individuals living alone were more likely to suffer more severe depression symptoms (29). Our results are partially the same as their study. However, we observed an opposite effect of the level of education compared to previous evidence. In a normal social environment, compared with the participants with higher levels of education, those with lower levels of education have the potential to undergo more challenges and stress (such as job loss, loan foreclosures, and other financial burdens), leading them to develop more negative emotional responses to stressors (30). In our study, the higher risk of depression for the residents with middle and high education than those with lower education may be related to the special environment of long-term stagnation of social communication where they were.

Notably, the medical staff were more inclined to express depressive symptoms, and the proportion of high depressive symptoms among medical staff (65.8%) was higher than that of residents (53.3%). Medical staff are busy at the front line of the epidemic almost daily, bearing the double physical and psychological burden. Therefore, we should give more care and tolerance to front-line medical staff to maintain their mental health.

Furthermore, we also found that the total PSQI score and each subscale of PSQI in the high depressive symptom group were higher than those in the low depressive symptom group. Huang et al. reported that the severity of depression will increase significantly when the cut-off of the PSQI global score reaches five (31). The relationship between sleep disorders and depression has also been reported in various nations and populations. Chronic sleep disorders have great influence on both physical and mental health. Long-term sleep disturbance in adults was associated with more severe depressive symptoms (32). Poor subjective sleep quality was strongly associated with various depression symptoms, causing poor quality of life among obese patients (33). The children with excessive daytime sleep tended to have parent-reported symptoms of depression and attention deficit (34). Our results are consistent with previous studies that have demonstrated strong associations between depression and other sleep disturbance like sleep latency and efficiency (35–38).

Logistic regression analysis revealed that the medical staff group and residents group shared two common risk factors of depression: “increasing internet usage time” and “daytime dysfunction.” Leménager et al. indicated that longer internet surfing might be associated with greater emotional disturbance, such as depression and current anxiety (39). We also found that “increasing internet usage time” and “daytime dysfunction” were nearly twice as likely to lead to more severe depressive symptoms in the medical staff group than in the residents group. Therefore, medical staff on the front line of the fight against the epidemic should better adjust their work and rest schedules, eliminate their dependence on mobile phones and the internet, and improve their energy during the day to avoid depression symptoms.

Nevertheless, our study has several potential limitations. First, it was a cross-sectional study, thereby weakening the dynamic analysis of depressive symptoms. Second, the questionnaires assessing mental health conditions used in our study were all self-rating scales that may not objectively represent the true prevalence of depression, anxiety, or sleep disorders. Diagnostic assessment tools will be added in further study. Finally, the results cannot be extrapolated to other countries and regions which adopt other levels of static management to control the COVID-19 pandemic.

The authors are also members of the doctors fighting against the COVID-19 pandemic. Just like most medical staff working on the front line, they have experienced the challenges of the 2 months, they waved goodbye to their families and rushed to the battlefield. Medical staff are also ordinary people and will get depressed once the stress is unbearable. The mental world of human beings is too fragile, and a person is normal because he is in a normal environment. In the special period of COVID-19 pandemic, we should learn to adjust our work and rest, reduce internet usage time, especially the medical staff who work hard at the front line.

## Conclusion

There are differences in the factors influencing depression symptoms between medical staff and residents during the 2022 city-wide temporary static management period to fighting against the COVID-19 pandemic in Shanghai. We should pay special attention to those with increased internet usage time and daytime dysfunction in medical staff working in a special environment such as the COVID-19 pandemic.

## Data availability statement

The raw data supporting the conclusions of this article will be made available by the authors, without undue reservation.

## Ethics statement

The studies involving human participants were reviewed and approved by the Ethics Committee of the Children's Hospital of Fudan University, Shanghai. Written informed consent for participation was not required for this study in accordance with the national legislation and the institutional requirements.

## Author contributions

YZ and YT contributed to the conception of the study, implementation of the survey, data analysis, chart production, and manuscript drafting. XB and QD carried out literature search and data analysis. CH, TL, and WZ provided assistance for the analysis with constructive discussions. JSu and JSh contributed significantly to the design of the study, data analysis, and finish the revised manuscript. All authors have read and approved the final manuscript. All authors contributed to the study conception and design.
